# The microbiome-product colibactin hits unique cellular targets mediating host–microbe interaction

**DOI:** 10.3389/fphar.2022.958012

**Published:** 2022-09-12

**Authors:** Walaa K. Mousa

**Affiliations:** ^1^ College of Pharmacy, Al Ain University, Abu Dhabi, United Arab Emirates; ^2^ College of Pharmacy, Mansoura University, Mansoura, Egypt

**Keywords:** colibactin, phage induction, genotoxicity, human microbiome, colorectal cancer, inflammation, colitis, DNA damage

## Abstract

The human microbiota produces molecules that are evolved to interact with the diverse cellular machinery of both the host and microbes, mediating health and diseases. One of the most puzzling microbiome molecules is colibactin, a genotoxin encoded in some commensal and extraintestinal microbes and is implicated in initiating colorectal cancer. The colibactin cluster was discovered more than 15 years ago, and most of the research studies have been focused on revealing the biosynthesis and precise structure of the cryptic encoded molecule(s) and the mechanism of carcinogenesis. In 2022, the Balskus group revealed that colibactin not only hits targets in the eukaryotic cell machinery but also in the prokaryotic cell. To that end, colibactin crosslinks the DNA resulting in activation of the SOS signaling pathway, leading to prophage induction from bacterial lysogens and modulation of virulence genes in pathogenic species. These unique activities of colibactin highlight its ecological role in shaping gut microbial communities and further consequences that impact human health. This review dives in-depth into the molecular mechanisms underpinning colibactin cellular targets in eukaryotic and prokaryotic cells, aiming to understand the fine details of the role of secreted microbiome chemistry in mediating host–microbe and microbe–microbe interactions. This understanding translates into a better realization of microbiome potential and how this could be advanced to future microbiome-based therapeutics or diagnostic biomarkers.

## Introduction

Trillions of microbes reside in the human gut interacting with each other and the outcome of their interaction affects human health and diseases (Sharon et al., 2014; Dhanaraju and Rao 2022; de Vos et al., 2022). This interaction is mediated by evolved small molecules, of which the vast majority are still dark matter (Wilson, Zha, and Balskus 2017; Zha et al., 2022). A few examples of microbiome secreted products are reported, each with a unique activity such as the microbial genotoxin colibactin (Wernke et al., 2020). Colibactin is a hybrid polyketide–non-ribosomal peptide product encoded in the genome of some Enterobacteriaceae that belong to the phylogenetic group B. Colibactin is implicated in causing colorectal cancer (CRC) by inducing a signature mutation in DNA. Isolation and structural elucidation of colibactin has challenged the scientific field for over 15 years because of its contact-dependent synthesis, minimal expression, and chemical instability. The bulk of the research study on colibactin is centered around revealing its structure, mainly through bioinformatic analysis, comparative metabolomics, and mutational studies (Tang et al., 2022). However, to appreciate the evolved function of colibactin(s) and other microbiome products, we must understand their ecological role in enhancing the competency and fitness of producers. There are some reports on the antimicrobial-like activity of colibactin, but most of the data come from observational or associational studies without revealing the underpinning mechanism of action (T. Faïs et al., 2016). In a pioneering study, the group of Dr. Balskus revealed that colibactin induces prophage activation on a wide scale in multiple phage–bacteria systems. This activity is mediated by its ability to damage DNA activating the SOS response. Moreover, induction of prophages affects prophage-encoded genes such as some bacterial toxins, which are crucial for their pathogenicity. This finding reveals another interesting role of colibactin in mediating microbe–microbe interaction and shaping the structure of gut microbes (Silpe et al., 2022; Tronnet et al., 2020).

Our knowledge of colibactin(s) is developing and there are previous reviews covering their prospective research period (Faïs et al., 2018; Bode 2015; Balskus 2015; Williams et al., 2020; Wernke et al., 2020; Tang et al., 2022). These reviews mainly focused on summarizing advances in understanding possible biosynthetic pathways, bioinformatic prediction of colibactin and precolibactin structures, and the mechanism of DNA mutation. In this review, we analyze the unique cellular targets of colibactin(s) mediating host–microbe interactions that shape gut microbiome structures and influence host’s health. We discuss the molecular mechanisms underlying the evolved function of colibactin in hitting multiple targets in both the eukaryotic and prokaryotic cell machinery. We propose a mechanistic model for the series of events leading to colibactin-induced cancer. This knowledge is central to better advancing the use of microbiome secreted products or genes as diagnostic biomarkers or therapeutic interventions.

## The latest development in understanding the biosynthesis and structure of colibactin(s)

### Prevalence and significance of colibactin gene cluster in prokaryotes

The first report on the colibactin cluster is dated back to 2006 when Nougayrède et al. (2006) reported the discovery of *Escherichia coli* strains belonging to the phylogenetic group B2 that can block mitosis, leading to megalocytosis and eventually cell death. The authors linked this weird activity to a genomic island that encodes cryptic molecule(s), named at this time colibactin (Nougayrède et al., 2006). Interestingly, this gene cluster is prevalent in gut microbes, in particular Group B of human-associated strains of *E. coli* and Enterobacteriaceae, including commensal and probiotic strains such as *E. coli* Nissle 1917, a commercial probiotic (Mutaflor) used to improve gastrointestinal inflammatory conditions such as ulcerative colitis (Schultz 2008). The Colibactin cluster is also sequenced from other non-human associated microbes such as *Pseudovibrio* sp. JE062, isolated from a marine sponge (Bondarev et al., 2013) and a novel strain of *Erwina oleae* sp. isolated from olive tree nots caused by *Pseudomonas savastanoi* (Moretti et al., 2011). The *Frischella perrara* PEB0191 is a commensal gut microbe in honey bees. The Crawford group showed that *F. perrara* PEB0191 produces colibactin mimics and induces DNA breaks similar to human microbiome strains (Engel, Vizcaino, and Crawford 2015). This widespread prevalence of colibactin or its homologs indicates its potential role in a symbiotic relationship, which likely confers beneficial outcomes to the host.

### Genomic organization of the colibactin cluster

**FIGURE 1:**
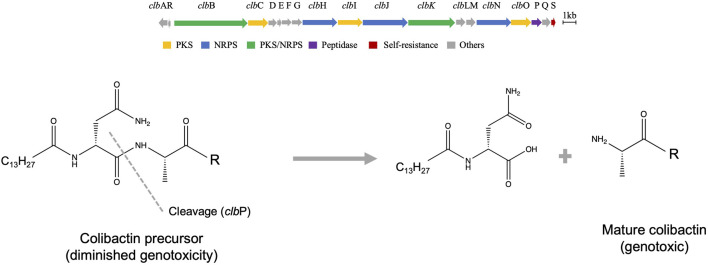
Genetic organization and possible biosynthesis of colibactin. **(A)** Organization of the biosynthetic genes cluster of colibactin. **(B)** Prodrug activation hypothesis overview shows the last required step for production of the genotoxin, which is catalyzed by peptidase enzyme.

The colibactin cluster encodes 54-kb hybrid nonribosomal peptide synthetase-polyketide synthase (NRPS-PKS) biosynthetic genes known as the *pks or clb* gene cluster (Figure 1). The cluster consisted of 19 genes, named alphabetically, clb A-S. These 19 genes encode three polyketide synthases (PKSs), three nonribosomal peptide synthases (NRPSs), two hybrids NRPS/PKS, MATE transporter, resistance gene, and other nine tailoring and accessory enzymes as per the latest update as reviewed (Tang et al., 2022). Intensive research reports confirm that all of these genes are essential for genotoxicity, except for clb M, S, and R (Nougayrède et al., 2006). Our current understanding of colibactin structure is shaped through understanding the organization of its biosynthetic genes, mutational studies, heterologous expression, and structure prediction using bioinformatics models. Over the last 16 years, there have been multiple hypotheses for the biosynthesis of colibactin, and the most recent is the prodrug activation theory (Figure 1) developed by the Balskus group (Brotherton and Balskus 2013; Balskus 2015). The genomic island of colibactin contains the *clbP* gene, which is a peptidase enzyme that shares structure/function homology with cleavage enzymes functioning in ribosomaly synthesized posttranslational modified peptides such as ZmaM in zwittermicin biosynthesis (Luo et al., 2011). This similarity is revealed by a plethora of confirmation analyses, including structure–activity relationship, crystallography, and mutagenesis (Cougnoux et al., 2012; Dubois et al., 2011). This fact leads Balskus and coworkers to hypothesize the prodrug activation theory for the biosynthesis of colibactin in which two NRPS modules (clbN and clbB) install the N-acyl-d-asparagine scaffold earlier in the biosynthesis, followed by clbP cleaving the amide bond and releasing the final active colibactin product (Brotherton and Balskus 2013). The Muller group added additional verification of the hypothesis by isolating the hypothesized prodrug from *E. coli*-Nissle 1917 (Bian et al., 2013). Further studies revealed that silencing of this peptidase enzyme (Δ clbP strain) facilitates the accumulation of precolibactins, which are more stable derivatives of the main genotoxic molecule. Δ clbP strains have a diminished genotoxic activity compared to wild type. Of note, we still lack more precise information on the genetic organization of colibactin-like clusters in other species and how the possible polymorphism might lead to functionally diverse molecules.

### Revealing the structure of colibactin derivatives (precolibactins)

**FIGURE 2:**
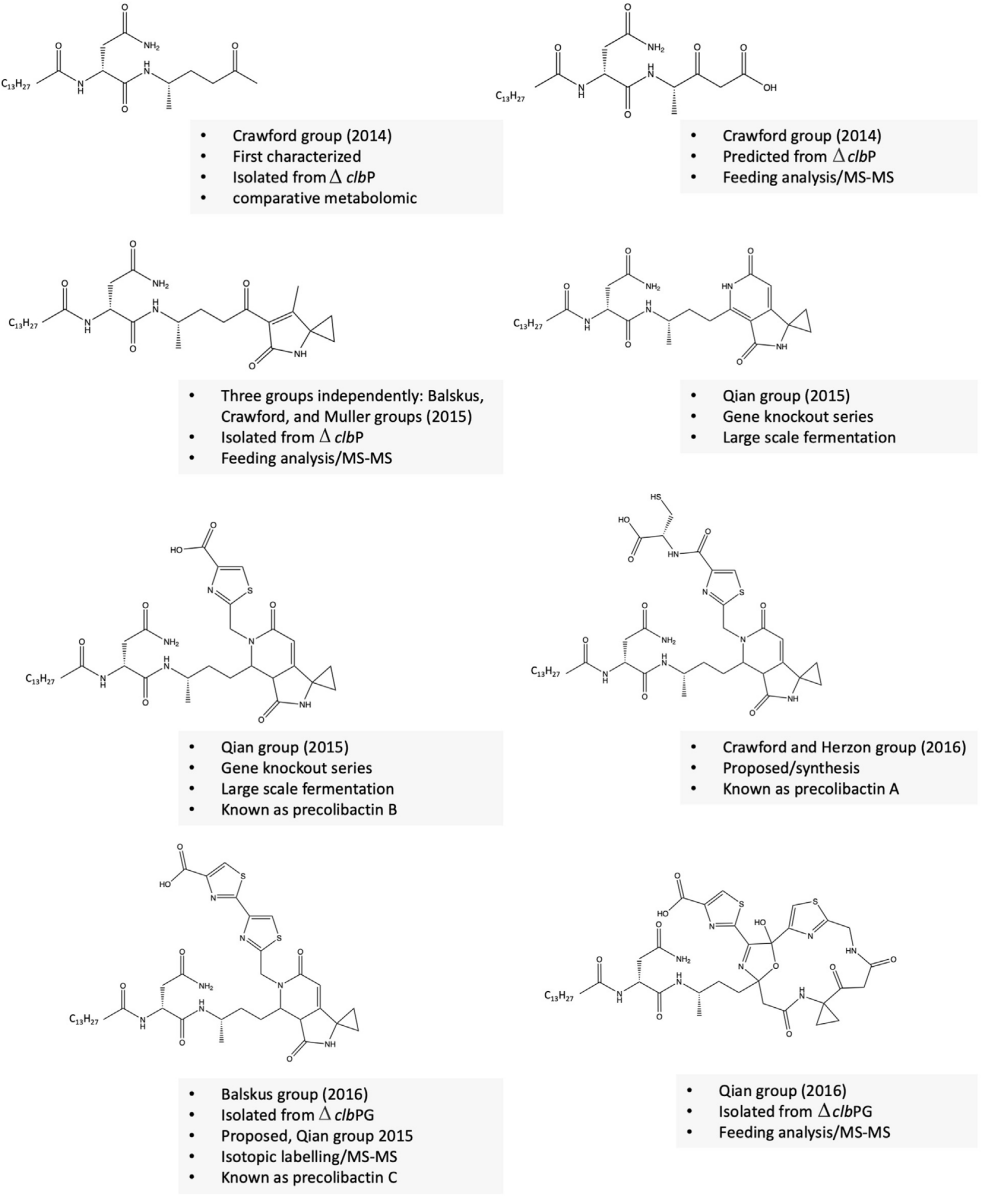
Selected examples of precolibactins with unique structures.

The precise structure of the colibactin molecule has remained unknown for over a decade. Purification of colibactin from native or heterologous hosts is challenging due to its minimal production upon fermentation, instability, and contact or inflammation-dependent gene expression. To overcome this challenge, several research groups developed alternative strategies to characterize the more stable derivatives or precursors of colibactin, named precolibactins (Vizcaino et al., 2014; Li et al., 2019; Vizcaino and Crawford 2015; Brotherton et al., 2015; Bian et al., 2015; Li et al., 2015; Li et al., 2016; Healy, Vizcaino, et al., 2016b). These strategies involve: 1) mutating the *clbP* gene, an essential enzyme required to produce active genotoxic colibactin molecules, and the generation of a series of other mutants; 2) heterologous expression of the mutated gene clusters; 3) comparative metabolomic analysis of mutants and wild-type strains; 4) tandem MS–MS fragmentation analysis coupled with isotopic feeding experiments; and 5) synthesis of potential precolibactin analogues. Following these approaches, around 40 precolibactins have been predicted, isolated, or synthesized. Some structurally unique examples of precolibactins are shown in Figure 2. Isolated or predicted precolibactins might be biosynthetically related, and for example, theoretically, precolibactin C could be biosynthesized from A via cyclodehydration as proposed (Healy 2017). Healy et al. proposed that precolibactins A-C may not be the precursors for colibactin as previously thought but rather alternative products produced only when the cibP is nonfunctional by a double cyclodehydration pathway (Healy et al., 2016a; Healy et al., 2016b). This hypothesis is based on some evidence that colibactin alkylates DNA by the formation of unsaturated imines, which could not be generated from the pyridone ring in precolibactins A-C (Healy et al., 2016a). Interestingly, some precolibactins share a structural similarity with yatakemycin and duramycin, mainly the aminocyclopropane moiety, which attacks DNA via nucleophilic ring-opening (Tichenor and Boger 2008). Precolibactin A weakly cross-links DNA *in vitro* while a precolibactin derivative lacking the spiro bicyclic structure was inactive (Vizcaino and Crawford 2015). Further evidence suggests that the two precolibactins isolated are products of the intact colibactin cluster.

### Revealing the structure of colibactin(s)

**FIGURE 3:**
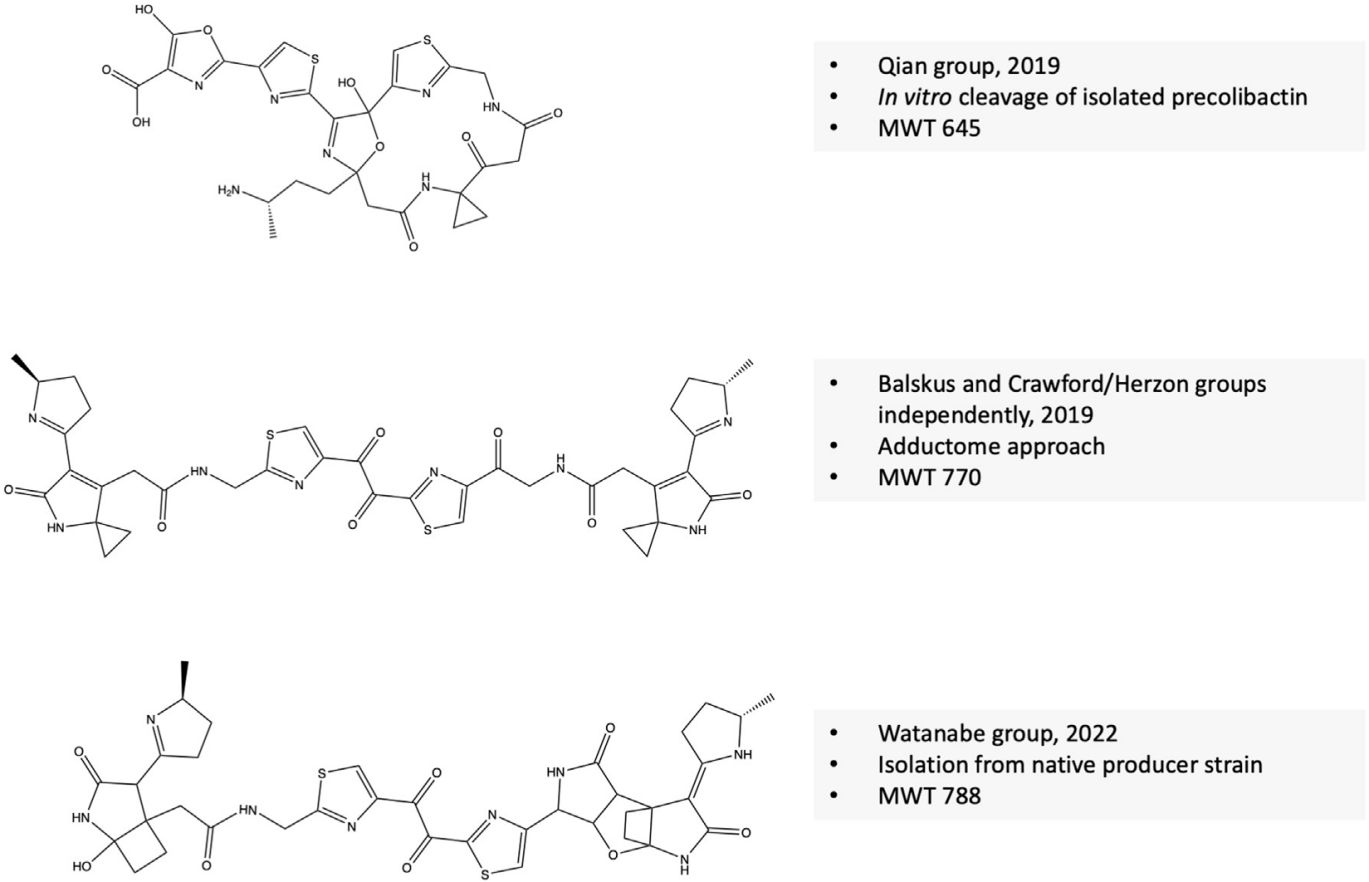
Confirmed structure of colibactin(s).

In 2019, the Qian group elucidated the structure of a colibactin molecule based on *in vitro* cleavage of an isolated precolibactin with an MWT of 969 to yield colibactin with an MWT of 645 (Li et al., 2019). Further evidence confirms that this structure is produced from the native PKS + strain. This evidence includes the presence of the product in the extract of native colibactin-harboring *E. coli* CFT073 as detected by MS–MS analysis and the ability of the compound to induce the characteristic DNA double-strand breaks (Li et al., 2019). In the same year, Crawford/Herzon and Balskus groups independently elucidated the structure of another colibactin molecule with an MWT of 770 and validated its genotoxic activity (Xue et al., 2019; Jiang et al., 2019). Crawford/Herzon predicted the structure by a DNA adductome approach followed by total synthesis. The Balskus group predicted the structure by studying *clbP*/*clbL* double mutants, labeling experiments, and an adductome approach. The clbL is predicted to encode amidase functions by hydrolysis of an amide bond; however, the authors validated that this enzyme is rather the final step in the production of intact precolibactin (Jiang et al., 2019). In 2022, the Watanabe group isolated a colibactin molecule with an MWT of 788 from the *pks + E. coli* strain-50, a strain isolated from colorectal cancer tissues that produces 26-fold more colibactin than Nissle 1917 (Hirayama et al., 2019; Zhou et al., 2021). Of note, the same group has developed an activity-based fluorescent probe to identify high colibactin producers from clinical samples 3 years before elucidating the first-isolation-based structure of colibactin (Hirayama et al., 2019). At first, the authors obtained two fragments of the compound whose structures are suggestive of spontaneous hydrolysis of the candidate molecule at the 1,2-diketone moiety as previously described by the Herzon group (Healy et al., 2019). Next, they managed to protect the compound from hydrolysis by adding *o*-phenylenediamine to convert the 1,2-diketone moiety into quinoxaline, which is not subjected to hydrolysis and isolated a few micrograms of colibactin sufficient for further NMR spectroscopic analysis (Zhou et al., 2021). The three revealed structures of colibactin are shown in Figure 3.

## Colibactin hits unique molecular targets in the eukaryotic cells

Our gut microbes secrete a plethora of molecules with diverse structures and functions. These molecules can either promote host health such as butyrate or trigger diseases. In addition to colibactin, several microbiome-derived molecules are noted as mediators of diseases, such as the autism-promoting molecule 4-ethylphenylsulfate and the gut genotoxin cyclomodulin (Hsiao et al., 2013; Buc et al., 2013). Colibactin might hit other targets in the eukaryotic machinery than what we currently appreciate. A study shows that some synthetic precolibactins exhibited antagonist activity on the mammalian brain receptors such as serotonin 5-hydroxytryptamine type 7 (5-HT7) *in vitro* (Vizcaino et al., 2014). Of note, 5-HT7 is expressed in dendritic cells. Studies show that 5-HT7 receptor antagonists such as SB-269970 decrease both acute and chronic inflammation in a mouse model of colitis (Kim et al., 2013). Synthetic precolibactin also showed antagonist activity against dopamine 5 receptors (Vizcaino et al., 2014). Dopamine receptors are reported to be located in the CNS and gut (Z. S. Li et al., 2006).

### Mechanistic insights into colibactin signature mutation

**FIGURE 4:**
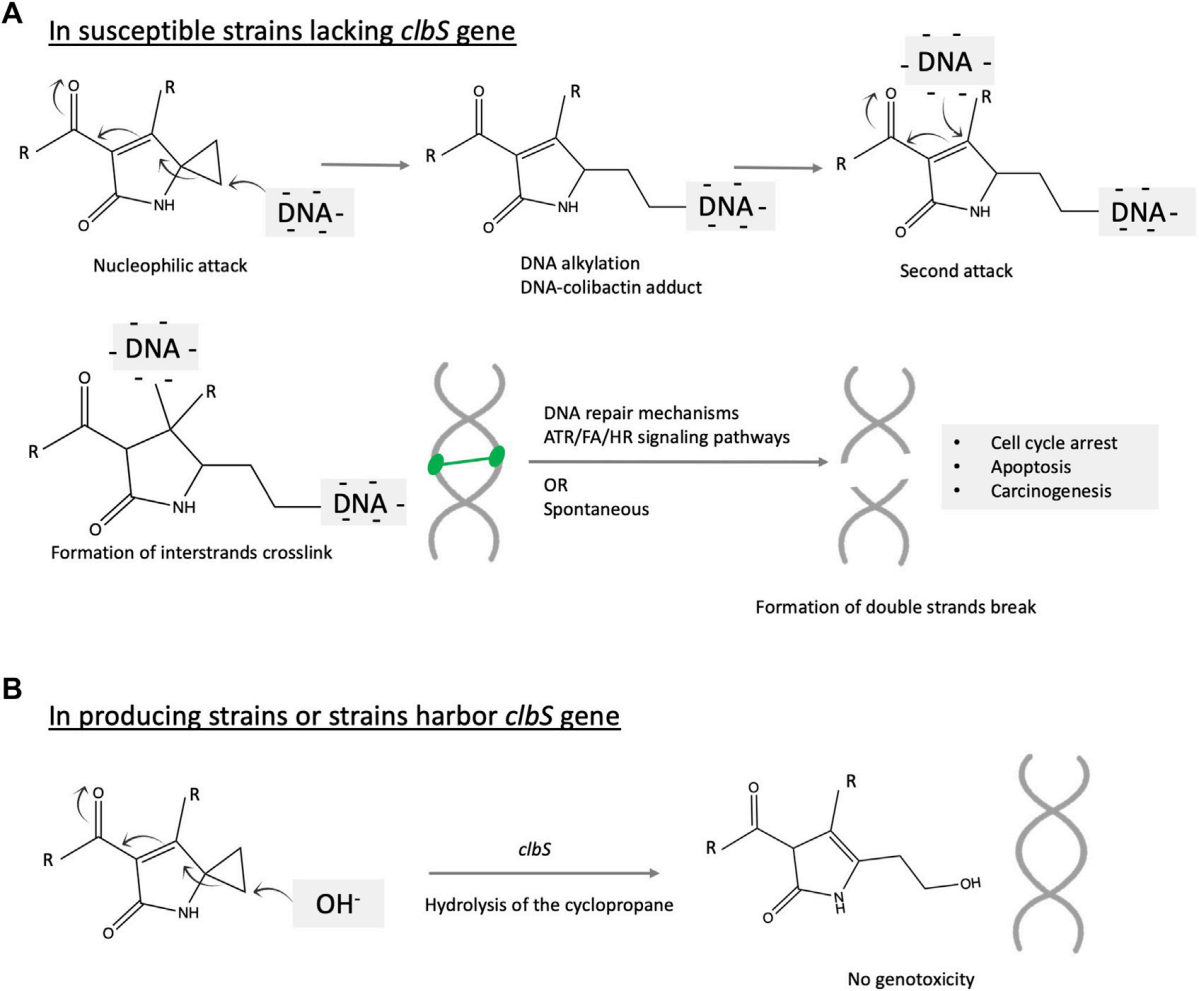
Illustration of the proposed mechanism of colibactin mutagenesis on the eukaryotic cell. The key structural scaffolds mediating this mechanism is the azospirobicyclic ring (warhead). The process is initiated by a nucleophilic attack on the electrophilic ring, which results in DNA alkylation and creates another electrophilic center subjected to a second alkylation reaction. Two alkylation events cross-link the DNA, which further activate multiple and complex DNA repair mechanisms including Ataxia telangiectasia mutated-and Rad3-related (ATR), Fanconi anemia (FA), homologous recombination (HR) signaling pathways. The DNA double strands break may be the product of the repair system or spontaneously formed. Accumulation of this mutation might initiate colorectal cancer.

Colibactin alkylates DNA in the eukaryotic cell leading to colorectal cancer. Overall, colibactin causes an inter-strand DNA cross-link, leading to double-strand breaks in the DNA triggering cell-cycle arrest and further to CRC. The mechanism of colibactin mutation is detailed below and illustrated in Figure 4. DNA double-strand breaks are prevalent in more than half of the patients suffering from inflammatory bowel diseases and CRC (Buc et al., 2013). The colibactin-DNA signature adduct is detected in both human and animal cells and *in vivo* in experimental animals (Wilson et al., 2019). A study conducted on mouse models of invasive carcinoma revealed that inoculation with *pks* ^*+*^ *E. coli* NC101 increases tumor and enables metastasis (Arthur et al., 2012). This tumor-promoting activity is diminished with the deletion of the colibactin gene cluster. However, this deletion does not affect the inflammatory status (Arthur et al., 2012). The DNA alkylation or formation of the covalent bond between the electrophilic warhead of colibactin (azospiro 2,4 bicyclic ring) and the nucleophilic DNA results in the formation of a colibactin–DNA adduct and creates a second electrophilic center that further reacts with DNA to form a cross-link. DNA alkylation is mediated by the electrophilic cyclopropane moiety of colibactin. Initially, colibactin forms an unstable adduct, which is considered a biomarker signature for colibactin (Wilson et al., 2019).

Cross-linked DNA adducts trigger multiple DNA repair signaling pathways, leading to the formation of double-strand breaks and further carcinogenesis (Figure 4). Another research study proposed an alternative mechanism of formation of this characteristic DNA mutation involving cupper-mediated oxidative damage (Li et al., 2019). DNA alkylating agents constitute a major class of chemotherapeutics that cause cytotoxic DNA damage and collateral mutagenic damage, leading to cell death. This damaging process is defended by several cellular mechanisms, including base excision repair, mismatch repair, and direct DNA damage reversal. The response to alkylating agents required delicate coordination between repair pathways and show much variability between cells and individuals as reviewed (Fu et al., 2012). Alkylating agents attack the nitrogen or oxygen atoms in the DNA nitrogenous base. Alkylating agents could be monofunctional if they only have one active moiety and can attack a single site on DNA or bifunctional if they have two reactive moieties and can bind to two separate bases and consequently crosslink DNA. In 2016, Healy et al. validated the hypothesis that colibactin alkylates the DNA by cyclopropane ring opening via the formation of unsaturated imines. The authors synthesized 13 structural mimics of colibactin and tested their DNA alkylation potential (Healy et al., 2016a). In 2019, Wilson et al. used chemical synthesis coupled with an untargeted DNA adductomic approach to reveal DNA alkylation in HeLa cell lines incubated with a colibactin-producing strain of *E. coli* (Wilson et al., 2019). The same reaction is observed *in vivo* in mice (Wilson et al., 2019). The authors reveal that this covalent DNA alkylation is mediated by the electrophilic cyclopropane moiety of colibactin (Wilson et al., 2019). Other data show that colibactin cross-links DNA. The study employed NMR spectroscopy and bioinformatics-guided isotopic labeling to characterize the colibactin warhead (Vizcaino and Crawford 2015). The synthetic colibactin mimic contains a spiro bicyclic structure and can cross-links duplex DNA *in vitro*. The authors proposed that colibactin alkylates DNA via a homo-Michael addition reaction, turning colibactin into a second Michael receptor. Thereafter, intramolecular Michael addition will generate a DNA cross-link (Vizcaino and Crawford 2015). This hypothesis is supported by pieces of evidence. First, is the observation that precolibactins do not form much of the higher molecular weight adduct product when reacting with linearized plasmid DNA. Second, synthetic colibactin inspired by the biosynthetic information showed that the cyclopropane ring could be a target for thiol nucleophile attack and is essential to shearing the DNA (Healy et al., 2016a). Further results based on gel electrophoresis reveal that, upon artificial dimerization, these colibactin mimics cross-link the DNA.

### Colibactin-induced events leading to inflammation and cancer

**FIGURE 5:**
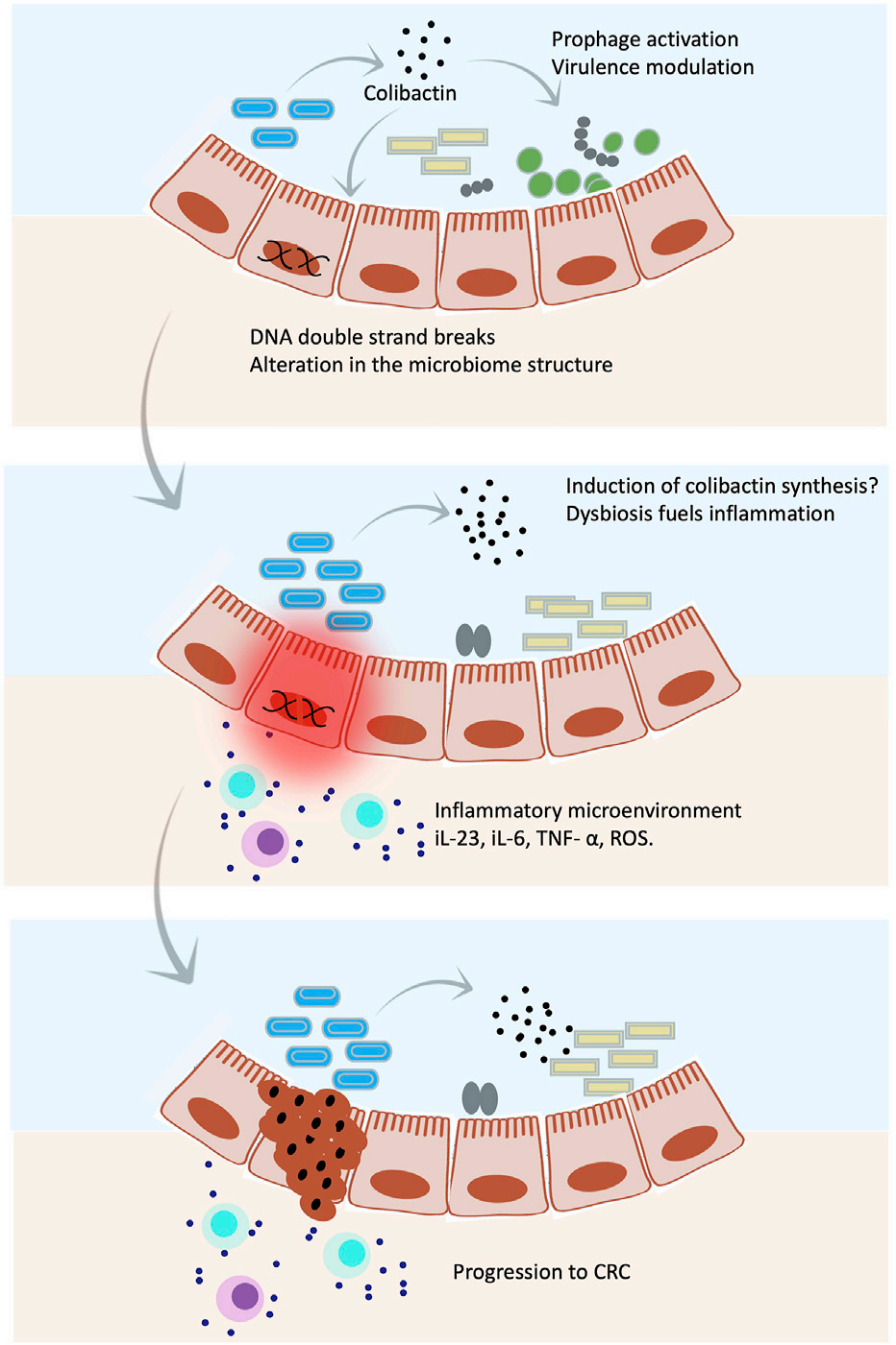
Colibactin hits targets in both eukaryotic and prokaryotic cells. Illustrations show an overall mechanism by which colibactin mediates host–microbe interaction. Colibactin shapes the gut microbial community by selective killing of some species through induction of prophages and/or stimulation of the virulence factor expression. Meanwhile, colibactin induces double strand breaks in host DNA. The altered microbial community together with the genetic abnormalities caused by colibactin triggers an inflammatory microenvironment, which might induce synthesis of more colibactin, leading to even greater damage that might result in CRC.

The interplay between genotoxic molecules such as colibactin and inflammation is not clear. It is speculated that DNA damage initiates tumor formation while inflammatory cytokines and cells promote it by creating a microenvironment that enables more DNA damage (Balkwill and Mantovani 2001; Ullman and Itzkowitz 2011) by, for example, inducing gene expression of genotoxic molecules. Previous studies show that microbial colonization enhances the development of cancer in mice that are genetically susceptible to inflammation (Ullman and Itzkowitz 2011). A shift in gut microbes was observed during inflammation, with unknown directionality, characterized by enrichment in genera of *E. coli*, *Akkermansia*, *Shigella*, and *Bacteroides* and a sharp reduction in Lachnospiraceae, Muribaculaceae, and Lactobacilli (Lang et al., 2020). Another study suggested that the microbial shift is associated with inflammation in the first place and not with cancer in the colitis-susceptible Il10^−/−^ mouse strain. Among the sifted species, *E. coli* showed a 100-fold increase in colitis (Ullman and Itzkowitz 2011). Additionally, *E. coli* NC101 causes profound colitis in germ-free mice with 80% of the subjects developing adenocarcinoma (Ullman and Itzkowitz 2011), again questioning the directionality of the association. Another study used *in vitro* 3-D cell culture models to examine the host factors that might contribute to susceptibility to colibactin-induced cancer. The authors reported that the presence of the expression of mucin genes in forming the mucous layer that adheres to the intestinal epithelia decreased the genotoxic effect of colibactin. Moreover, the removal of the mucous layer in other models restored the genotoxicity (Reuter et al., 2018). Since inflammation is associated with degradation of the mucous layer and a decrease in the tight junction protein, also known as “leaky gut” that might be another factor to add to the colibactin–inflammation–cancer equation. Inflammation also allows leakage of microbes and other microbial products, not only colibactin, which flares up an even more intense immune response (Ahmad et al., 2017; Kidane et al., 2014). Of note is that the microbial composition in the presence of colibactin is altered, which might promote the abundance of pro-inflammatory bacteria. The interplay between colibactin-induced inflammation and cancer is illustrated in Figure 5.

## Colibactin hits targets of the prokaryotic cells controlling microbiome structure

### The genomic organization of the colibactin cluster suggests a potential antimicrobial activity

Microbes are known to produce microbial toxins such as antibiotics to kill competing microbes and gain significant ecological competency. To protect themselves, the toxin-producing strains mostly harbor self-resistance genes. The presence of a self-resistance gene in the colibactin cluster suggests that colibactin is not only affecting cellular target in the human cell but also the bacterial cell. The biosynthetic gene cluster of colibactin ends with a resistance gene encoding a 170-amino acid protein named *clbS. clbS* encodes a cyclopropane hydrolase, resulting in the ring-opening of the DNA alkylating scaffolds, conferring self-resistance to producing strains (Tripathi et al., 2017) (Figure 4B). This gene is not only present in colibactin-producing strains but also in some other strains that lack the entire colibactin cluster, suggesting that this gene is transferable within the bacterial community by horizontal gene transfer to gain an ecological advantage. *clbS*-like genes with at least 50% sequence similarity have been found in members of the human gut microbiome, namely, *Escherichia albertii*, *Kluyvera intestini*, and *Metakosakonia* sp. and the honeybee microbiome, namely, *Snodgrasella alvi* (Silpe et al., 2022). Heterologous expression of these four clbS-like genes resulted in attenuated DNA damage and phage lysis when co-cultured with a colibactin-producing strain, suggesting that acquisition of clbS provides immunity against colibactin-induceddamage. Interestingly, *clbS* protects against colibactin-specific induction of prophages but provides no protection against other inducing agents such as MMC. This resistance is intra-cellular and is not shared between cells (Silpe et al., 2022). Other studies show that deletion of the *clbS* gene does not kill the strain but its growth will be dependent on the DNA repair mechanism (Bossuet-Greif et al., 2016).

### Molecular targets behind colibactin activity on prokaryotes

Colibactin belongs to the PKS/NRPS natural products, which include many antibiotics such as daptomycin and β-lactams (Walsh 2004). Earlier studies suggested that colibactin might have antibiotic-like activity but the precise mechanism was not clear. A study showed that *E. coli* harboring a colibactin cluster exhibits growth inhibition against multi-resistant *Staphylococcus aureus* in the agar diffusion method and growth competition assay. This activity is observed in 95% of the tested strains, while the ΔclbP *E. coli* lost this activity (T. Faïs et al., 2016). The authors reported that this activity requires live culture, suggesting it is an inducible trait. Another study reported that inoculation of pregnant mice with a colibactin harboring strain resulted in a decreased abundance of firmicutes and a significant alteration in microbial diversity in pups, especially after 35 days of birth (Tronnet et al., 2020). Interestingly, the authors reported increased activity in DNA repair pathways, suggesting that colibactin not only modulates the structure of gut microbes but also their function (Tronnet et al., 2020). Another research study shows that the colibactin biosynthetic gene (*clbA*) might affect siderophore biosynthesis enhancing its producer fitness (Martin et al., 2013).

In 2022, the Balskus group revealed an interesting mechanism beyond the observed activities of colibactin in shaping the microbial population (Silpe et al., 2022). They discovered that colibactin-induced damage activates SOS signaling pathways, leading to the induction of prophages exerting lethal action on their host bacteria. The authors show that the effect of colibactin on prophages extends to a wide range of phages residing in phylogenetically distinct bacteria, including *Salmonella typhimurium*, *Staphylococcus aureus*, *Citrobacter* rodentium, and *Enterococcus* faecium (Silpe et al., 2022). Interestingly, there is a significant increase in Shiga toxin production upon co-culture of *C. rodentium* (harboring Stx genes) with *pks* + *E. coli* (Silpe et al., 2022)*.* Induction of prophages enables selective lethal action against other members of the microbial community, providing an elegant ecological advantage and enhancing the competitiveness of the producing strain.

### Molecular mechanism of prophage induction following DNA damage in the host

**FIGURE 6:**
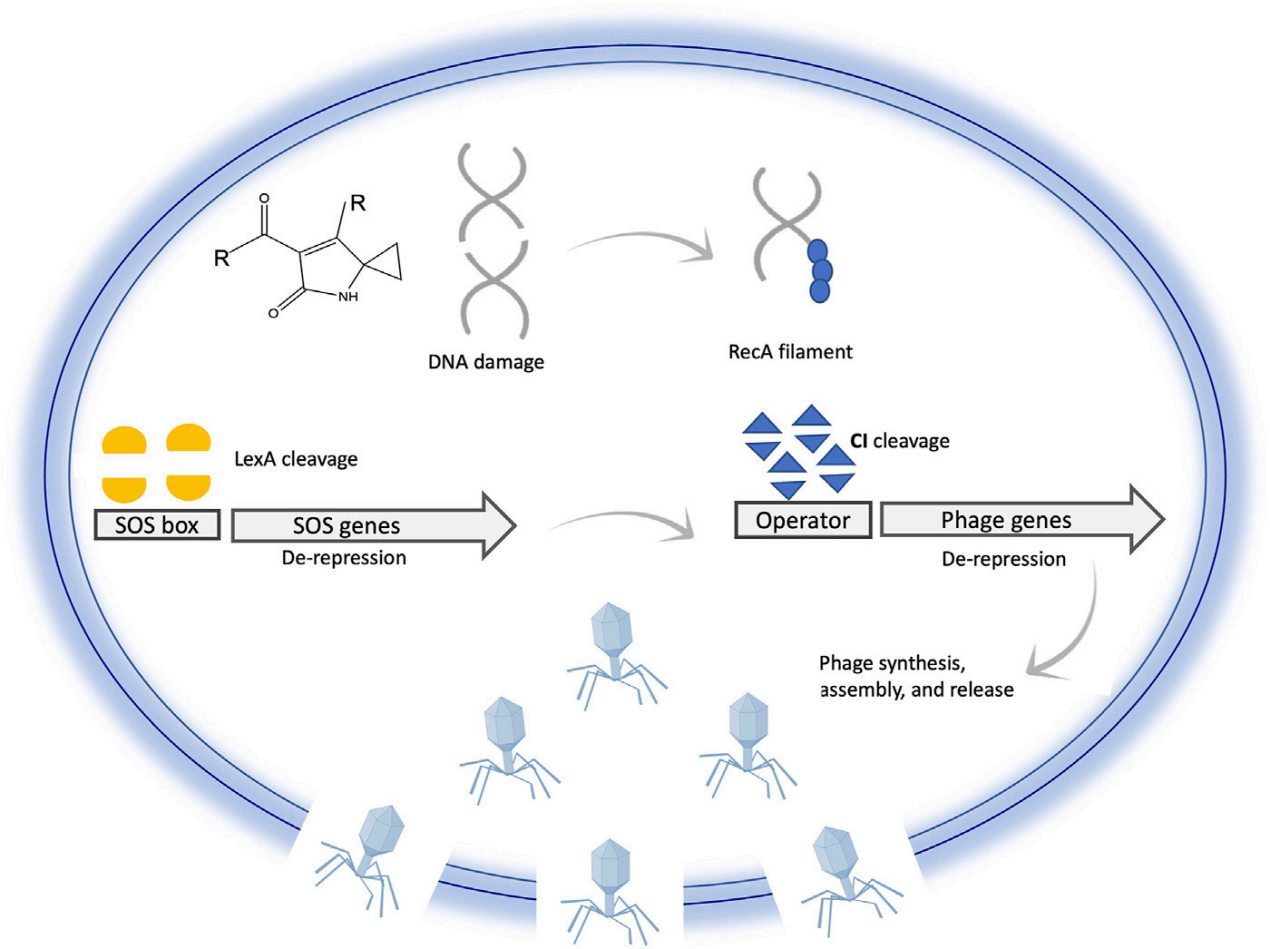
Illustration of prophage activation following DNA damage inside a bacterial cell. DNA damage induces cleavage of phage repressor genes mediated by SOS signaling. Cleavage of the phage repressor leads to exciton of prophage from the bacterial chromosome, synthesis of phage-related genes and proteins, assembly, cell lysis, and phage release. This illustration is simplified to communicate the main idea; for example CI binds to two distant regions and not only to one site as shown for simplicity of the illustration.

Lytic activation of prophages in bacterial lysogens is normally induced by DNA damage, such as after exposure to UV radiation or DNA damaging agents such as mitomycin C (MMC) (Lee et al., 2006; Humphrey, Stanton, and Jensen 1995). In addition to DNA damage, other internal and external stimuli might lead to prophage induction, such as pH, heat, reactive oxygen species (ROS), or even spontaneous induction (Nanda, Thormann, and Frunzke 2015). Maintenance of lysogeny is tightly controlled by the activity of the λ CI repressor proteins. Two dimers of CI bind to the operator regions of phage genes and suppress their expression (Hochschild 2002; Little, Shepley, and Wert 1999). The CI protein is a molecular switch that controls lysogeny in lambda phages. CI binds to operator regions of phage genes and represses their expression while inducing its own expression. DNA damage activates the master regulator RecA, resulting in de-repression of SOS genes mediated by cleavage of their transcriptional repressor LexA (Gimble and Sauer 1989; Little and Michalowski 2010; Thomason et al., 2021). Activation of SOS genes and RecA leads to the cleavage of the CI protein resulting in the de-repression of phage genes to enter the lytic cycle (Hochschild 2002; Little, Shepley, and Wert 1999). This molecular mechanism is simply illustrated in Figure 6. Genetic mutations affecting dimerization of lambda repressor increase the rate of its cleavage, while repressor dimer stabilized by covalent disulfide bond resists cleavage (Gimble and Sauer 1989). An exception is the prophages in *Salmonella* genomes, which uses another inductive strategy based on the use of anti-repressor molecules that bind the dimer without cleavage, leading to its dissociation from the DNA. Production of these anti-repressors is under the direct control of LexA (Lemire, Figueroa-Bossi, and Bossi 2011).

### Prophages contribute to the ecological fitness of harboring strains

Viral genes make up to 20% of microbial genomes, either as prophage-like elements, phage remnants, or fully prophages that could be induced into the lytic life cycle (Wang et al., 2010). These prophages contribute to the ecological fitness and virulence of the lysogens. Reports show that lysogens can use prophages as a survival strategy (Bossi et al., 2003; Gama et al., 2013). For example, in a mixed culture, *Salmonella enterica* induces its prophages, leading to the killing of some self-cells while the others are converted back to lysogens. The released prophages are then used as a weapon to kill competitors although it results in some self-destruction. This strategy will also benefit the phage by spreading its genes (Bossi et al., 2003; Gama et al., 2013). Another example is the induction of prophages in *Streptococcus pneumoniae* to wipe off *S. aureus* via the production of hydrogen peroxide-inducing SOS genes. Induction of SOS leads to activation of prophages in *S. aureus*, which are mostly lysogens while S. *pneumoniae* are immune, giving S. *pneumoniae* an ecological advantage to dominate (Selva et al., 2009; Pericone et al., 2003). A study shows that deletion of nine cryptic prophages from *E-coli* suppresses fitness by rendering the bacteria more susceptible to sublethal concentrations of β-lactam and quinolone antibiotics. Moreover, this deletion rendered the bacteria less able to form biofilms and more prone to damage caused by acid or osmotic pressure (Wang et al., 2010). A study shows that prophages enable biofilm formation in *Pseudomonas aeruginosa*, contributing to its virulence and. when cells die in the biofilm, the prophage is converted to a super infective form (Rice et al., 2009). Biofilm is a group behavior in which bacterial cells adhere to one another and solid surfaces using a sticky matrix made of extracellular proteins, carbohydrates, and DNA molecules (Madsen et al., 2012). The formation of biofilm is a virulence strategy that helps enable bacteria to resist antibiotics and host immune defense. Prophage induction in a biofilm will lead to the accumulation of extracellular DNA promoting horizontal gene transfer and enhancing diversification of the microbial community within the biofilm (Molin and Tolker-Nielsen 2003). The role of prophages on biofilm formation and further consequences on host fitness is reviewed (Nanda, Thormann, and Frunzke 2015). The production of toxins is a crucial virulence trait for pathogens (Brown et al., 2006). Some toxins are encoded by bacteriophages, including cholera toxin (Ctx) and Shiga toxins (Stx), required for the virulence of *Vibrio cholerae* and *E. coli*, respectively (Waldor and Mekalanos 1996; Neely and Friedman 1998). For example, Shiga toxin (Stx) in enterohemorragic *E. coli* enables bacterial cells to attach and colonize the gut epithelia (Robinson et al., 2006). Similarly, some cells of *E. faecalis* V583 and *Streptococcus mitis* induce prophages to express phage genes to help the remaining cells adhere to human platelets, leading to systemic infection (Matos et al., 2013; Seo et al., 2010). This regulatory activity of microbiome-secreted molecules has been noted before. For example, quinolone antibiotics increase the production of Stx toxin via activation of prophages via SOS response (Pleguezuelos-Manzano et al., 2020). Co-culture of colibactin with *Citrobacter rodentium* increases the production of Stx (Silpe et al., 2022). Another interesting activity of colibactin was reported by Marcq et al. (2014). Driven by the association between pks^+^ *E. coli* strains and septicemia, the authors investigated the effect of colibactin-harboring strains on lymphopenia and sepsis. The results show that colibactin-producing *E. coli* causes the characteristic double-strand break in the DNA of lymphocytes, leading to an exaggerated lymphopenia and subsequent low survival rate from bacteremia in mice (Marcq et al., 2014).

## Conclusion

Colibactin demonstrates the potential of microbiome secreted chemistry to hit multiple targets spanning prokaryotic and eukaryotic cell machinery. It is interesting to speculate that colibactin gives the producing species an ecological advantage in occupying specific niches such as the human gut by controlling other competitive microbes. Understanding how precisely colibactin-producing strains alter gut microbiome composition and their further indirect impact on human health and diseases seems exciting. Of note, we still do not appreciate all the microbiome species that we host in our bodies or their cryptic genes and encoded chemistry. Revealing the microbiome products that exert microbial control and affect population dynamics will help to advance better strategies to tackle the antibiotic resistance crisis. Microbiome chemistry is certainly a new uncharted Frontier for understanding mediators of human conditions and developing innovative therapeutics or diagnostic biomarkers. For example, delivering colibactin mimics or genes to induce the lethal killing of a cancer cell or as an antimicrobial for life-threatening biofilm infection.

### Is colibactin a harmful molecule?

Host-associated microbes produce molecules that help to increase their ecological fitness and competitiveness and to establish a long-term, mostly symbiotic, relationship with the host. From an ecological perspective, we can imagine colibactin as a beneficial microbial product that is produced to shape the microbial community and confer beneficial traits to the host, such as decreasing inflammation as long as the mutualistic relationship is established. Evidence for this hypothesis came from the research on Nissle 1917. The efficacy of probiotic *E-coli* Nissle 1917 in treating colitis and preventing further remission is even comparable to that of mesalazine, the gold standard drug in the treatment of colitis (Kruis et al., 2004). The mechanism of action involves modulation of cytokine expression. Interestingly, further investigation on Nissle 1917 activity suggests that its beneficial anti-inflammatory activity is dependent on the expression of the colibactin biosynthetic pathway (Olier et al., 2012). Knockout strains that lost the ability to express the colibactin cluster not only lost the anti-inflammatory activity but also resulted in more inflammation (Olier et al., 2012). Moreover, inflammation is thought to be a triggering signal for further expression of the colibactin gene cluster. Chronic inflammation and accumulation of DNA damage will eventually lead to cancer development. The question now is, do these microbiome strains, or more specifically colibactin, initiate cancer or evolved to combat cancer and act as an immunomodulin based on the activity profile of *E. coli* Nissle 1917? One can postulate that it produces colibactin to kill transformative cells at the beginning of inflammation in a similar way to chemotherapeutic agents and more inflammation triggers more synthesis of the mysterious molecule(s). This bidirectional dependency is puzzling and poses an outstanding question on the directionality of microbiome diseases association and warns of the urgent need for a holistic deep understanding of the microbiome secreted chemistry evolved to hit multiple cellular targets under very tight and complex regulation resulting in a variety of negative and positive outcomes for the host. Another interesting hypothesis is that the colibactin biosynthetic cluster encodes or supplies precursor molecules with immunomodulatory activity as originally proposed (Olier et al., 2012). Support for this hypothesis comes from the strong association between colitis and microbial dysbiosis, so it is interesting to speculate on the role of colibactin in shaping microbiome structure and whether it may restore the balanced composition to the pre-colitis status. However, the members of Enterobacteriacea are often considered proinflammatory, and some reports show significant enrichment in Enterobacteriacea with the onset of gastrointestinal inflammatory conditions (Garrett et al., 2010) and the dominant presence of adherent-invasive strains of *E. coli* such as LF82 (Carvalho et al., 2008), belonging to the same phylogenetic group as Nissle 1917, although it lacks the colibactin genomic cluster. Taken together, our knowledge of microbiome mediators and their evolved function is still in its infancy. Despite the explosion of research on the microbiome, the diversity and richness in species of the microbiome, their cryptic genes, and secreted molecules are still a dark matter.

### A proposed model for colibactin–microbe–host interaction and health outcome

**FIGURE 7:**
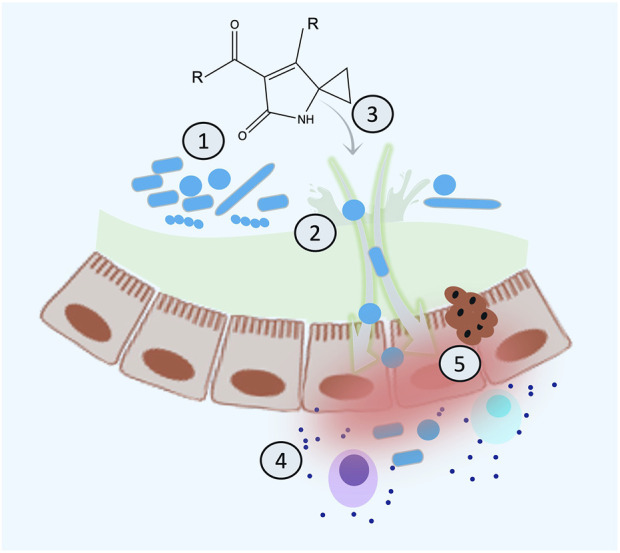
Proposed mechanistic model for the sequence of the events of colibactin-induced cancer. **(A)** Colibactin induces dysbiosis, **(B)** dysbiosis results in inflammation and leaky gut, **(C)** colibactin diffuses inside the cell resulting in DNA damage, **(D)** chronic inflammation increases the load of colibactin inside the cell and more DNA damage, and **(E)** accumulating DNA damage leads to CRC.

Host-associated microbes produce toxic molecules with the primary function of killing competitors and gaining an ecological advantage to dominate a particular niche. However, these toxins might also hit molecular targets in the host. Considering that the priority function of colibactin is microbial related, we can propose that the reasonable order of events leading to CRC might be as follows: 1) colibactin shapes microbial composition in the gut to modulate inflammation, especially during microbial dysbiosis; 2) chronic dysbiosis triggers inflammation, which leads to leaky gut; 3) colibactin diffuses inside the eukaryotic cell and causes DNA damage eliciting the repair mechanism; 4) chronic inflammatory microenvironment further advances the microbial dysbiosis, and gut permeability resulted in more diffusion of colibactin to eukaryotic cells (or maybe an induction of its synthesis); and 5) under high load of colibactin, accumulation of DNA damage might occur leading to colorectal cancer (Figure 7). This situation gets worse with a genetic predisposition to inflammation or defect in the DNA repair pathways. Considering this model, chronic microbial dysbiosis and inflammation are crucial for colibactin-induced colorectal cancer and might serve as a risk factor to predicting CRC, especially if this is combined with genetic disorders in genes related to the DNA repair pathways or immune function. This understanding is not only helpful in predicting risks but also in taking preventative measures such as implementing an anti-inflammatory diet for high-risk groups to decrease the incidence of CRC.
